# 2182. Expression of the MadR regulon in *Enterococcus faecalis* OG117 is influenced by the presence of the LiaS Histidine Kinase of the LiaFSR pathway

**DOI:** 10.1093/ofid/ofad500.1804

**Published:** 2023-11-27

**Authors:** Samie A Rizvi, Sara I Gomez Villegas, Diana Panesso, Cesar A Arias, William R Miller

**Affiliations:** Houston Methodist Hospital, Houston, Texas; Brigham and Women's Hospital - Harvard Medical School, Boston, Massachusetts; Houston Methodist Research Institute, Houston, Texas; Houston Methodist and Weill Cornell Medical College, Houston, TX; Houston Methodist Research Institute, Houston, Texas

## Abstract

**Background:**

The MadRS pathway is a two-component signaling (TCS) system which mediates resistance to antimicrobial peptides (AP) and AP-like antibiotics in E. faecalis (**Fig. 1**). The system is composed of a transmembrane histindine-kinase, MadS, which is responsible for the phosphorylation of the transcriptional response regulator, MadR. This leads to increased expression of the madR regulon, containing *madG*, *madL*, and *dltA,* which provide resistance against APs and AP-like antibiotics. Interestingly, deletion of *madS* does not result in loss of expression of *madG*, suggesting an alternative mechanism of phosphorylation of MadR independent of MadS. We hypothesize that LiaS, of the LiaFSR pathway, may phosphorylate MadR in the presence of APs and AP-like antibiotics.

**Figure 1.**
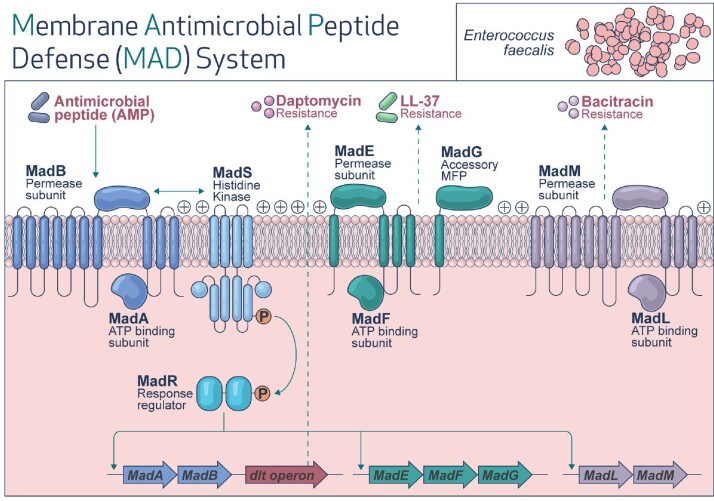
MadRS initiates a regulatory response to defend against antimicrobial peptides.

In the presences of APs, the MadS Histidine Kinase phosphorylates the MadR response regulator, leading to transcription of downstream genes madAB, madEFG, madLM and the dlt operon.

**Methods:**

Wild type (WT) *E. faecalis* OG117, OG117FΔ*madS*, and OG117Δ*liaS*Δ*madS* were grown to mid-exponential phase in tryptic soy broth. Each strain was grown either with or without ½ of the minimum inhibitory concentration (MIC) of bacitracin (BAC), an inducer of the MadRS system. RNA was isolated, and expression of *madG* was evaluated by qRT-PCR relative to the control strain, OG117, without bacitracin exposure. Values were normalized to the *gyrB* “housekeeping” gene. Fold-change was calculated using the Pfaffl method. Differences in gene expression were determined using two-way ANOVA with Tukey’s test for multiple comparisons.

**Results:**

Both the control strain, OG117, and the OG117Δ*madS* mutant exhibited a BAC MIC of 32 mg/L. The BAC MIC for OG117Δ*liaS*Δ*madS* decreased to 2 mg/L. In the presence of BAC exposure, OG117Δ*madS* had a statistically significant increase in *madG* expression (p< 0.001) as compared to OG117Δ*madS* without BAC exposure (**Fig. 2**). In contrast, OG117Δ*liaS*Δ*madS* displayed no significant change in *madG* expression when exposed to bacitracin (**Fig 2**).

**Figure 2.**
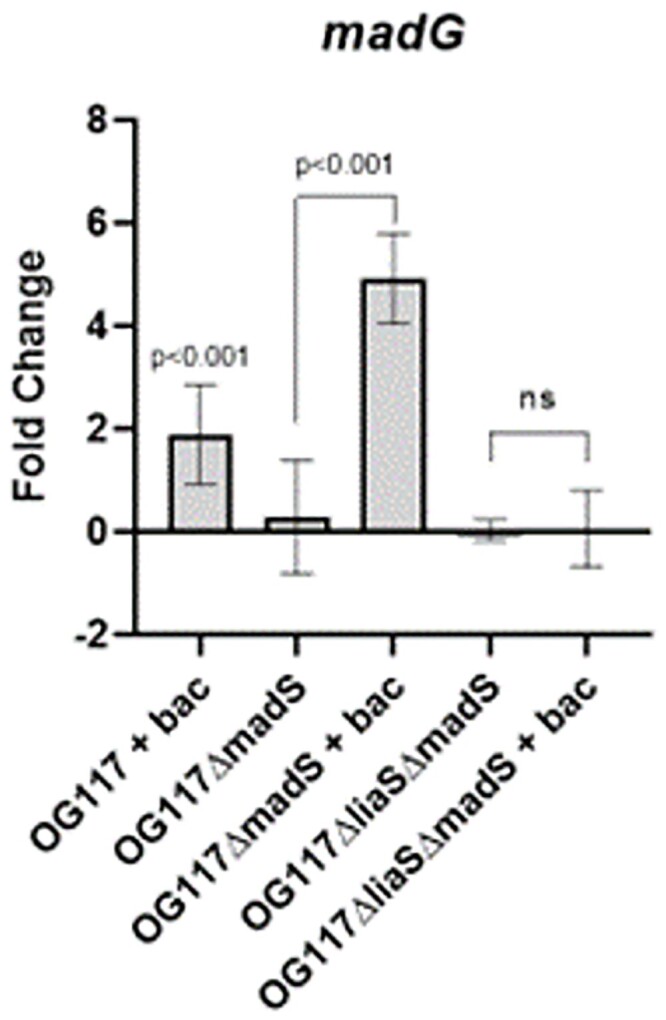
Gene expression of madG in OG117, madS, and liaSmadS knockout strains.

**Conclusion:**

In the absence of a functional MadS histidine-kinase there is still transcriptional upregulation of *madG*. This effect is reversed with the deletion of both LiaS and MadS. This suggests that the LiaS histidine-kinase may lead to MadR dependent activation of the MadRS pathway in *E. faecalis* OG117.

**Disclosures:**

**William R. Miller, M.D.**, Merck: Grant/Research Support|UpToDate: Honoraria

